# Selection and Optimization of Carbon-Reinforced Polyether Ether Ketone Process Parameters in 3D Printing—A Rotating Component Application

**DOI:** 10.3390/polym16101443

**Published:** 2024-05-20

**Authors:** Raja Subramani, Praveenkumar Vijayakumar, Maher Ali Rusho, Anil Kumar, Karthik Venkitaraman Shankar, Arun Kumar Thirugnanasambandam

**Affiliations:** 1 Center for Sustainable Materials and Surface Metamorphosis, Chennai Institute of Technology, Chennai 600069, India; sraja@citchennai.net (R.S.); arunkumar.t@citchennai.net (A.K.T.); 2 Department of Mechanical Engineering, Amrita Vishwa Vidyapeetham, Amirtapuri 690525, India; praveenkv@am.amrita.edu; 3 Lockheed Martin Engineering Management, University of Colorado, Boulder, CO 80308, USA; maru4732@colorado.edu; 4 Department of Mechanical Engineering, Kamala Nehru Institute of Technology, Sultanpur 228118, India; anilk@knit.ac.in; 5 Centre for Flexible Electronics and Advanced Materials, Amrita Vishwa Vidyapeetham, Amritapuri 690525, India

**Keywords:** additive manufacturing, Material Extrusion, carbon-reinforced Polyether Ether Ketone, MCDM, Fuzzy-AHP-TOPSIS, process parameter selection, optimization

## Abstract

The selection of process parameters is crucial in 3D printing for product manufacturing. These parameters govern the operation of production machinery and influence the mechanical properties, production time, and other aspects of the final product. The optimal process parameter settings vary depending on the product and printing application. This study identifies the most suitable cluster of process parameters for producing rotating components, specifically impellers, using carbon-reinforced Polyether Ether Ketone (CF-PEEK) thermoplastic filament. A mathematical programming technique using a rating method was employed to select the appropriate process parameters. The research concludes that an infill density of 70%, a layer height of 0.15 mm, a printing speed of 60 mm/s, a platform temperature of 195 °C, an extruder temperature of 445 °C, and an extruder travel speed of 95 mm/s are optimal process parameters for manufacturing rotating components using carbon-reinforced PEEK material.

## 1. Introduction

Additive manufacturing (AM) technology, as an alternative to subtractive techniques within the manufacturing industry, constructs objects layer by layer and garners substantial interest across various domains. Within this paradigm of 3D printing, objects are generated by directly segmenting their geometries from digital formats and feeding this data into the 3D printing machine. The literature has delineated seven distinct processes within additive manufacturing, categorized based on the raw materials employed and the printing methodologies utilized. The Material Extrusion (MEx) process is the simplest and most prevalent 3D printing technique [1,2,3]. The Material Extrusion (MEx) process involves using thermoplastic polymer filaments to manufacture the end product. Optimization of MEx process parameters is deemed crucial for product printing, as it directly influences the control of printing machine operations, printing duration, and mechanical properties of the final product. This optimization factor fine-tunes input data for the 3D printing machine following the feed design. Key parameters include printing speed, travel speed, extruder temperature, and printing pattern [4,5,6]. Previous studies have extensively investigated the selection of process parameters for carbon reinforcement. This section highlights some of the notable research papers in this area. A comprehensive explanation is also provided regarding the reasoning behind the meticulous selection and optimization process of carbon-reinforced polymers, coupled with using mathematical programming techniques that previous researchers have explored or employed to select the final product of rotating components.

Lu et al. [7] conducted a study in which they produced carbon-reinforced PEEK composites in various ratios and examined their mechanical properties. Their findings revealed that blending carbon fibre with PEEK at a ratio of 20 weight % significantly impacts the mechanical properties. Therefore, in the present research, carbon was incorporated into the PEEK material at a lower ratio of 10 weight %. Chidambaram et al. [8] studied the process parameters of layer thickness and printing speed to optimize PEEK material’s hardness and wear characteristics. The investigation determined that a printing speed of 20 mm/s and a layer thickness of 0.15 mm resulted in superior hardness and wear behaviour. Sivakumar et al. [9] explored the application of machine learning algorithms to optimize the selection of process parameters for manufacturing final fusion cages using PEEK material. Parameters such as layer height, printing temperature, printing speed, infill density, built orientation, and line width were carefully considered in the investigation. The research determined that an optimal range for layer thickness falls between 0.1 mm and 0.3 mm, printing temperatures range from 370 to 410 degrees Celsius, printing speeds vary from 10 mm/s to 50 mm/s, infill densities range from 40 percent to 100 percent, the optimal built orientation spans from 0 degrees to 90 degrees, and line widths range from 0.1 mm to 0.3 mm.

Kechagias et al. [10] have investigated to ascertain the porosity levels of components produced through 3D printing process parameters. This exploration involved considering various FDM process parameters, including infill rate, infill density, built orientation, part orientation, printing speed, bed temperature, environmental conditions, and nozzle temperature. Ultimately, the study revealed a significant impact of printing parameters on mechanical loading and porosity. Jiang et al. [11] have explored process parameters employing the Taguchi method to attain high-strength products utilizing PEEK material. The Taguchi L9 experiment encompassed crucial process variables such as printing speed, layer thickness, printing temperature, and extruder strand width. The culmination of the investigation revealed that for the production of high-strength final products using PEEK material, optimal parameters include a printing speed of 5 mm/s, a layer thickness of 0.1 mm, an extrusion strand width of 0.4 mm, and a printing temperature of 395 °C.

Liu et al. [12] have investigated the optimization of process parameters for bone manufacturing with PEEK material. Parameters such as layer thickness and infill density were carefully considered in this study. The findings revealed that the optimal process parameters for PEEK material are a layer thickness of 0.43 mm and an infill density of 55 percent. While various studies have explored process parameter selection for PEEK material, there is limited literature on the selection of process parameters for carbon-reinforced PEEK materials. Particularly in applications involving rotating components, no research has yet determined the process parameters for carbon-reinforced PEEK material.

### Thermoplastic Polymers in Rotating Component Application

Polymers have been increasingly utilized to manufacture rotors in various systems, enhancing the efficiency of blowers and siphons and improving cost-effectiveness [13,14,15]. Industry favours these materials for their affordability, ease of manufacturing, water resistance, and adaptability [16,17]. The extrusion process for polymer materials can involve a variety of raw materials, including powders, granules, filaments, and resins, depending on the manufacturing method and raw material used. Thermoplastic polymers such as Acrylonitrile butadiene styrene (ABS) [18], Polylactic Acid (PLA) [19], Polyethylene Terephthalate (PETG) [20], Polyether ether Ketone (PEEK) [21], and Polyphenylene sulfide (PPS) [22] have been utilized in the production of rotating parts. For instance, in a miniature organic rankine cycle (mORC), Hernandez-Carrillo et al. [23] investigated using an ABS impeller. In their study, the traditional load of the impeller was reduced to enhance the mORC’s efficiency. Operating conditions for the impeller included an input temperature of 55 °C, a pressure of 4 Bar, an exit temperature of 44.9 °C, and a pressure of 2.5 Bar, with a rotational speed of 36,000 rpm. After considering the operating conditions and the factor of safety (FoS), which is the ratio of yield strength to the maximum equivalent stress, the ABS impeller met the required performance criteria. One significant advantage of using this polymer was its ability to enable mass production of mORCs by reducing the cost of impeller manufacturing using ABS. The impeller’s isentropic efficiency was also estimated to be between 76% and 86% based on their simulations for the penta-fluor propane working fluid. However, one limitation noted was the restricted operation of ABS at temperatures above 89.9 °C.

Pavlovic et al. [24] assessed the mechanical properties of ABS for use in impeller pump construction, demonstrating its reliability for impeller manufacturing. Similarly, Polak [25] confirmed the hydraulic qualities of an ABS impeller for a spiral diffusive siphon, noting an efficiency increase at an alternating velocity of 2950 rpm. The remarkably smooth surface of the ABS impeller was identified as a key factor contributing to this efficiency improvement. A thermoplastic material called PLA, derived from renewable resources such as sugar beet or maize starch, offers biodegradability and composability as some of its key attributes. Compared to other polymers, PLA’s low cost, environmental friendliness, biocompatibility, and suitable physical and mechanical properties make it a preferable choice. PLA has been used to manufacture impellers for pumps [26,27], compressors [28], and maritime applications [29]. Despite the availability of both PLA and ABS, these two thermoplastic types have been extensively studied as materials for pump impellers. ABS is often chosen due to its higher resistance to stress compared to PLA, which exhibits high fragility in comparison [30].

Birosz et al. [31] examined PLA impellers for blowers, focusing on PLA’s tensile strength, creep behaviour, and bending characteristics. Creep testing results indicated that PLA behaved more like a poorly cross-linked elastomer under low loads, with the creep curve maintaining stability over time. This study contributed to the advancement of PLA as a potential material for long-term use. PLA is a thermoplastic that is semi-crystalline. Pump and mORC applications have utilized PLA impellers. For the creation of pump sharp edges, this polymer was chosen on the grounds of its brilliant water resistance and biodegradability. The PLA impeller was concentrated by Odetti et al. [32] for a Pump Jetting Module (PJM) application. A PLA impeller tried for this application showed satisfactory qualities with a speed of turn of 1200 rpm and a push of 14 N.

Zywica et al. [33] conducted a study on plastics and identified them as potential materials for ORC framework impellers. This investigation utilized two thermoplastic polymers: PPS and PEEK. The impeller was subjected to 120,000 cycles per minute. The study concluded that PEEK polymer is well-suited for impeller production based on simulation results pertaining to heat resistance, chemical resistance, tensile strength, and thermal expansion. Rotational components, such as gears, shafts, and impellers, are crucial parts of various mechanical systems where durability, wear resistance, and chemical resistance are paramount. Carbon-reinforced PEEK is a high-performance thermoplastic compared to neat PEEK and offers exceptional mechanical properties, including high strength, stiffness, and fatigue resistance, making it an ideal material for such applications. However, the literature gap regarding CF-PEEK in impeller applications remains unexplored.

#### Mathematical Programming Technique

The three primary categories for optimization techniques in operations research are statistical methods, stochastic techniques, and mathematical programming techniques. Using a numerical value, the statistical technique selects the best option from the experiment data. Response surface methodology (RSM), Taguchi, and design of experiment (DOE) are a few instances that may be used conventionally. Optimization via approximation theories is known as a stochastic approach. Selecting and optimizing using mathematical programming involves building quantitative numerical models based on qualitative data and solving them. This study employs a mathematical programming technique called multicriteria decision-making (MCDM). Multicriteria decision-making has the structure of selecting the best choice from multiple options based on multiple criteria for a single objective. Also, the commonly used statistical optimization tools (Taguchi method, Design of Experiment, RSM method etc.) and optimal solutions are found after multi-stage algorithms. Optimization, maximization, or minimization is the final result, but as far as the multicriteria decision-making technique is concerned, selection and optimization will be available as the final result. In this, multiple choices will be analyzed using a pair-wise matrix, depending on the objective based on each criterion. Finally, the choices are sorted, and the ranking is given. This helps prioritize and choose choices according to the situation, and a higher ranking indicates maximization or minimization. The accompanying previous research utilized MCDM approaches to select problems in additive manufacturing. In order to choose the best additive manufacturing technology for a customized product in Industry 4.0, Malaga et al. [34] used the hybrid MCDM method. Raja et al. [4] applied the MCDM strategy to choose the appropriate additive manufacturing machine from the 3D printer client’s perspective, utilizing the Fuzzy TOPSIS technique. The neutrosophic best-worst MCDM technique was utilized by Ghuge et al. [35] to pick the provider of additive manufacturing services. The hybrid MCDM technique was used by Chandra et al. to choose the sustainable additive manufacturing concept for solving environmental challenges [36].

Raja et al. [5] applied the analytical hierarchy process (AHP) MCDM technique to choose the best Material Extrusion machine in view of the additive manufacturing machine clients’ opinions. Subramani et al. [6] explored the different MCDM strategies and accentuated the choice examination issues in additive manufacturing. For that reason, numerous MCDM systems are utilized to address choice challenges in additive manufacturing. The Fuzzy-AHP-TOPSIS strategy again utilizes a five-guide rating framework to assess the algorithm and choices. Moreover, five similar choices were utilized in the examination level headed, and the reason for the simple linguistic terms is that the TOPSIS approach was applied in this research. Comprehensive details will be presented in the forthcoming section.

This study aims to identify the optimal cluster of Material Extrusion (MEx) process parameters for producing high mechanical properties in end products using carbon-reinforced Polyether Ether Ketone (CF-PEEK) filament. This filament is not typically employed in the fabrication of rotating components, specifically impellers. The objective is to achieve superior mechanical properties in a shorter production time. To address these research gaps, this research outlines Part two describes the tests necessary for rotating component applications, along with the material and method. Detailed results and discussions are presented in the final section. 

## 2. Materials and Methods

In this study, we investigate the application of carbon-reinforced polyetheretherketone (PEEK) in additive manufacturing processes to enhance the mechanical properties of manufactured components. The selection of the optimal material and process parameters is crucial to achieving high-quality prints with the desired properties. To address this, we employ the fuzzy analytical hierarchy process (AHP) in conjunction with the technique for order of preference by similarity to the ideal solution (TOPSIS). These methods allow for a comprehensive evaluation and selection of materials and process parameters, considering multiple criteria such as mechanical properties, cost, and environmental impact. This combined approach provides a systematic and effective way to optimize the additive manufacturing process for carbon-reinforced PEEK, contributing to the advancement of polymer-based additive manufacturing technology.

### 2.1. Assumptions of the Research

This study aims to identify the optimal cluster of printing parameters for carbon-reinforced PEEK based on the analysis of tensile, flexural, and morphology data results. To achieve this objective, the following assumption is made:

- This study considers multiple process parameters that can affect the mechanical properties of the final product. For instance, if the extrusion temperature decreases while the printing speed is high during the fabrication of a product, the final result may be compromised if the raw material does not solidify as expected. Similarly, variations in mechanical characteristics can occur if the printing speed is high, the extruder travel speed is slow, and the infill amount fluctuates.
- The evaluation framework is based on the results of the available data. Criteria 1 (C1) corresponds to the observation of ultimate tensile strength (UTS), Criteria 2 (C2) corresponds to the observation of Young’s modulus, Criteria 3 (C3) corresponds to the observation of ultimate flexural test, and Criteria 4 (C4) corresponds to the observation of surface defects. Notably, the options mentioned are labelled explicitly as Sample 1 to Sample 5, rather than Alternative (A1, A2, A3, A4, and A5).
- As reported in previous literature, printing parameter values range from the minimum to maximum values for both PEEK and carbon-reinforced PEEK, refer Table 1.
- Furthermore, the infill printing parameter was set to a normal “line” as per standard practice, although it can be adjusted to accommodate other infill designs such as hexagonal or triangular patterns. Each of the alternatives mentioned represents a distinct set of parameters (referred to as a cluster of process parameters), as outlined in Table 1.

### 2.2. Material Extrusion Printing

The MEx process transforms a product’s design from CAD models or other design software into its physical form. Modern slicing technology (Flash Forge 6.0 Zhejiang Flashforege 3D Technology Ltd., Zhejiang, China) is utilised. STL (Standard Triangle Language) files are divided into layers with varying printing parameters before slicing. This sliced data is then inputted into the MEx machine (Zortrax Endureal 3D printer, Olsztyn, Poland) for printing. The extruder moves the carbon-reinforced PEEK filament from the spool to the melting zone, where it is melted and deposited layer by layer on the build platform according to the input data. Previous studies have investigated polymer composite filaments to analyze the effects of carbon reinforcement on their properties.

#### Material Extrusion Process Parameter

The fused deposition method (FDM) process parameters have been significantly enhanced, exerting a notable influence on the durability of the final products. Numerous researchers have explored a wide array of controllable characteristics to achieve desired part features, with many convergent on a set of critical factors. The process parameters under scrutiny in this study are delineated below. The internal structure of the printed component is formed using the infill pattern printing technique, offering a variety of filling patterns, including cubic, diamond, triangle, hexagonal, honeycomb, linear, and line. Honeycomb is the optimal inner structure material for superior mechanical qualities, albeit with longer production times [37]. Consequently, employing the appropriate infill pattern without altering the build time or other print quality settings is strongly recommended.

The infill density indicates how much material is printed on a specific component and directly impacts its quality. A fully infilled density, when build delays are inconsequential, and an optimal infill density, when material customization and build times are feasible, yield the best mechanical strength [38]. At lower infill densities, a solid cross-section has a minimal effect on material failure. The volume of material deposited along the machine’s vertical axis in a single pass determines the layer height of a Material Extrusion machine.

Before commencing AM, the 3D CAD model must be sliced, as shallow slice heights prolong the construction process, while high slice heights result in a pronounced staircase effect and poor surface quality. Mechanical strength increases with layer height due to decreased void density [39]. Print speed, which influences build time and print quality, along with material deposition quality, plays a crucial role [40]. A faster print speed increases tensile strength due to the speed at which successive layer surfaces bond [41].

The temperature condition denotes the temperature at which the model material is heated by the system, controlling both the amount of molten material extruded from the nozzle and the platform temperature. Extrusion temperature refers to the temperature maintained in the heating head nozzle of the FDM prior to filament extrusion, enhancing fusing within and between layers as the temperature rises [42].

**Figure 1:**
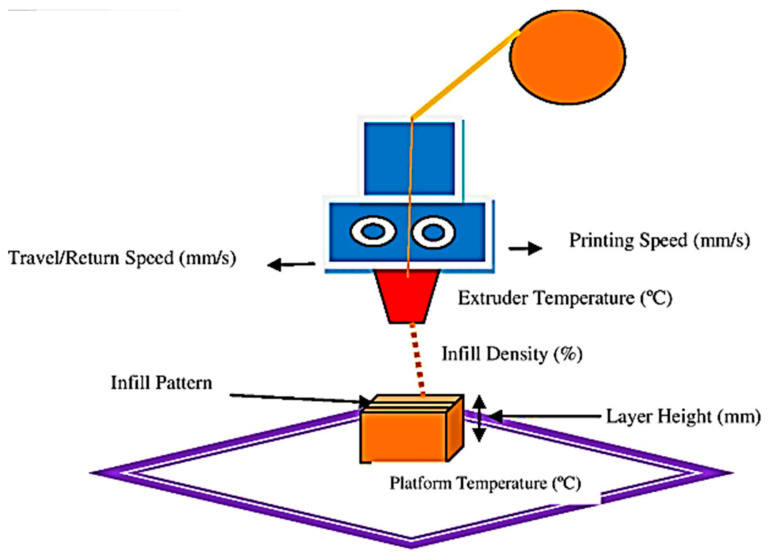
Schematic of MEx Process Parameters.

The speed at which an object is built also depends on the extruder’s velocity and the parts’ orientation in the build bed relative to the X, Y, and Z axes. The Z axis moves toward a part’s thickness or height while parallel to the build platform. Part travel speed impacts both mechanical and surface characteristics [43]. Figure 1 presents a schematic diagram illustrating the process parameters of the Material Extrusion method in additive manufacturing.

**Table 1:** Process parameters are taken for printing CF-PEEK [7,8,9,10,11,12,21,33].

Process Parameter	Infill Pattern	Layer Height (mm)	Print Speed (mm/s)	Platform Temperature (°C)	Extruder Temperature (°C)	Travel Speed (mm/s)	Infill Density (%)
Alternative 1	Line	0.30	30	180	430	80	55
Alternative 2		0.25	40	185	435	85	60
Alternative 3		0.20	50	190	440	90	65
Alternative 4		0.15	60	195	445	95	70
Alternative 5		0.10	70	200	450	100	75

**Figure 2:**
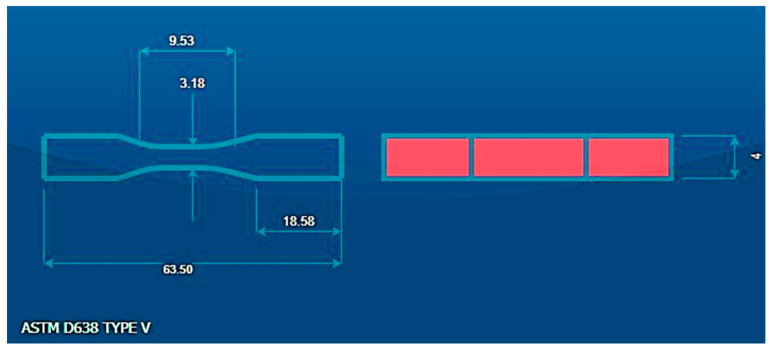
Experimental specimens.

**Figure 3:**
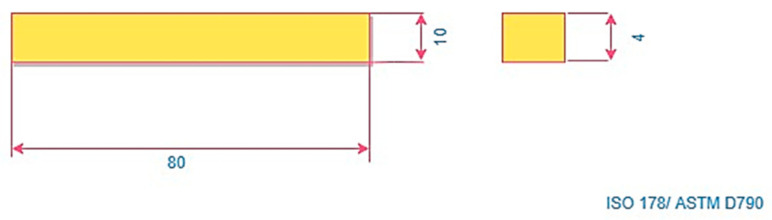
Experimental specimens [45].

Figure 2 illustrates the geometric standard for the ASTM D638 type V tensile test [44], while Figure 3 portrays the geometric standard for the ISO-178 flexural test of printed specimens produced on an FDM machine, with parameters outlined in Table 1.

### 2.3. Fuzzy AHP-TOPSIS

**Figure 4:**
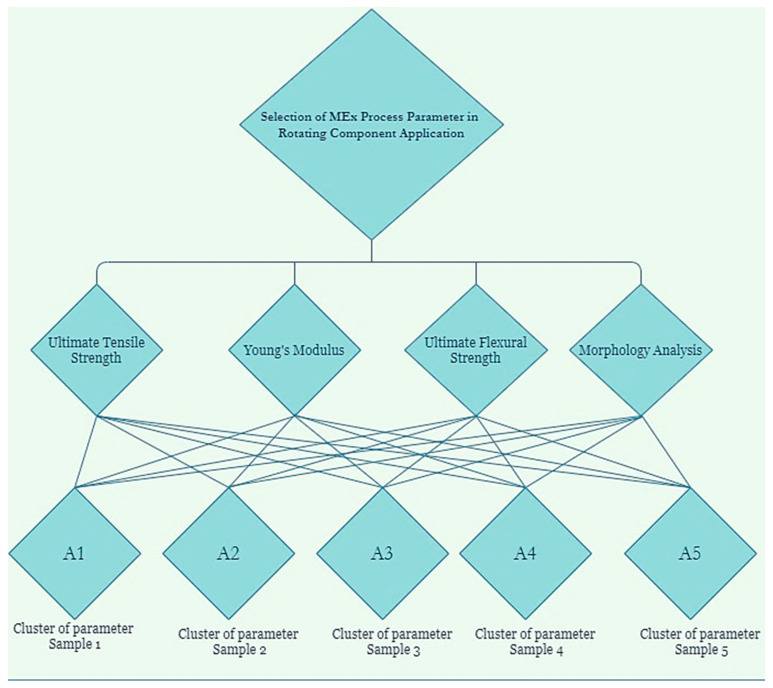
Concept of this research.

Fuzzy-AHP-TOPSIS depends on the rule of tracking down the best option, in contrast to the biggest mathematical separation from the negative ideal solution and the briefest mathematical separation from the positive ideal solution. By assessing how intently the choices look like the best response, Fuzzy-AHP-TOPSIS additionally decides the most ideal choice. The Fuzzy-AHP-TOPSIS approach is applied as continues in this study article:

The Analytic Hierarchy Process (AHP), created by Thomas Saaty in the 1970s, is expanded upon by the Fuzzy Analytic Hierarchy Process (AHP). The Analytic Hierarchy Process (AHP) is a systematic approach to handling complex decisions using a hierarchical structure to represent the decision criteria at various levels of abstraction. It is extensively used in many business, engineering, and healthcare domains for decision-making. Pair-wise comparisons of the alternatives and decision criteria are performed using the Analytic Hierarchy Process (AHP) to create priority scales determining the ultimate choice. Nonetheless, decisions made by decision-makers may not always be exact or free from uncertainty in many real-world scenarios. The Fuzzy AHP, which uses fuzzy logic to handle imprecise and uncertain information, was created to overcome this limitation. By employing linguistic variables such as “very low,” “low,” “average,” “high,” and “very high” to characterize the relationships between criteria and alternatives, fuzzy logic enables the representation of ambiguous and subjective judgments [5,42,46]. Instead of using exact numerical values for pair-wise comparisons, fuzzy numbers are used in the Fuzzy AHP. A membership function that gives each linguistic variable a degree of membership defines these fuzzy numbers. The priority scales for the criteria and alternatives are then obtained by combining the comparisons using fuzzy arithmetic operations. Figure 4 illustrates the fundamental concept and hierarchical structure of the research framework’s Fuzzy Analytic Hierarchy Process (AHP) Technique for Order of Preference by Similarity to the Ideal Solution (TOPSIS) method.

**Figure 5:**
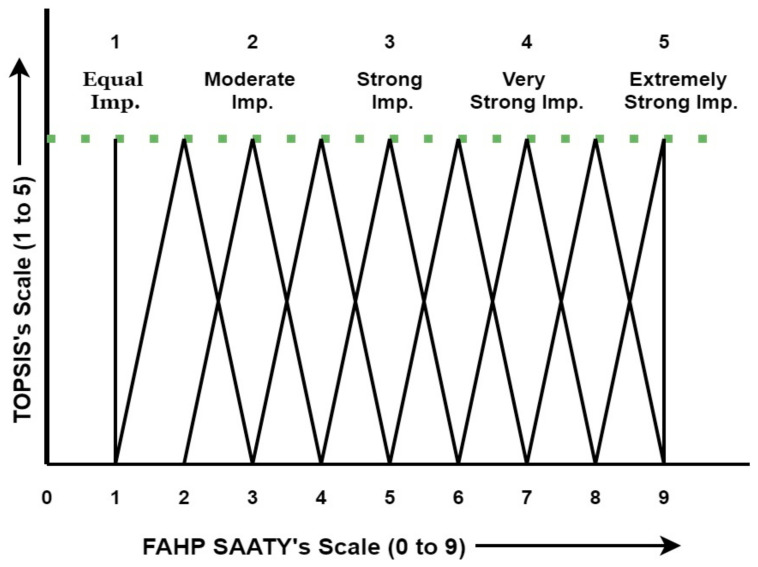
Scale conversion using the triangular membership function.

In this paper, the triangle membership function is utilized. The accompanying image (μå) is ordinarily used to address a Fuzzy value. Figure 5 depicts the triangle membership function in this exploration.

μå (*X*) = Å = (1, 2, 3) (1)

As per Equation (1), the lowest, middle, and upper fuzzy numbers in triangle membership functions are 1, 2, and 3 separately [4].

This study employs the linguistic terms outlined in Table 2. These terms are consistent with the previously described fuzzy numbers derived from the same triangle membership function.

This study employs various integrated decision-making methods alongside systematic group decision-making. Specifically, linguistic terms are utilized to construct a collaborative decision matrix encompassing alternatives and criteria. Subsequently, the corresponding fuzzy values are adjusted accordingly, as depicted in Table 3.

In the initial stage of implementing the Fuzzy Analytic Hierarchy Process (AHP) and Technique for Order of Preference by Similarity to the Ideal Solution (TOPSIS) method, the first step involves constructing a Pair-wise matrix using the provided data. Subsequently, the process normalises the decision matrix, constituting the second step. Following this, the third step involves distinguishing between beneficial and non-beneficial criteria. Using these identified criteria, the subsequent step involves determining the fuzzy positive ideal solution and the fuzzy negative ideal solution. Once these solutions are established, the differences between them are computed, leading to the calculation of the closeness coefficient. This detailed procedure is exclusively elaborated upon in the discussion section, which focuses on the results.

### 2.4. Mechanical Testing

Tensile testing was conducted using the Tinius Olsen H10KL machine, employing each set of parameters for three specimens, totalling 15 specimens across five parameter sets. Flexural tests were employed to evaluate both the composites’ flexural strength and the failure surface’s characteristics. Through these tests, the flexural stress of each specimen was measured across three trials. Criteria 3 was established to evaluate the different samples based on the average flexural test results and the flexural modulus findings. To ensure consistent testing conditions, the span length of the specimen was multiplied by four times its thickness, maintaining an equal distance from the bottom solid support.

### 2.5. Morphology Analysis

The sample prepared with revised set of printing parameter was analyzed using FESEM (Field Emission Scanning Electron Microscopy). This analysis revealed microstructural imperfections in the samples. Subsequently, morphology analysis was conducted to evaluate criteria 4, assigning points on a linguistic scale ranging from 1 to 5.

## 3. Results and Discussion

As per prior research, ultimate tensile strength (UTS) significantly influences tensile outcomes. The values for Young’s modulus and ultimate tensile strength (UTS) are presented in Table 4 and Table 5, respectively. Trials A, B, and C were performed on each specimen, and their average results for assessing the criteria using linguistic terms were calculated for both Young’s modulus and UTS.

According to the observations in Table 4, A4 exhibits the lowest average Young’s modulus value, whereas Sample 3 demonstrates the highest average value.

The observation of Table 5 indicates that A2 exhibits the highest average UTS value, whereas A5 demonstrates the lowest value. As per the linguistic term scale, A2 was rated very high (VH) and A5 was rated Very Low (VL).

According to Young’s modulus, based on the available data, Alternative 3 exhibits the highest strength at 3.68 GPa, securing the top position, followed by Alternative 1 at 3.54 GPa, ranking second, Alternative 2 at 3.19 GPa, ranking third, Alternative 5 at 3.08 GPa, ranking fourth, and Alternative 4 at 3.01 GPa, ranking fifth. Consequently, the TOPSIS Linguistics scale attributes a significance score of 5 to high-strength Alternative 3, indicating its utmost importance, while assigning a score of 1 to low-strength Alternative 4, reflecting its comparatively lesser significance. 

Consistent with this, Alternative 2 exhibited the highest strength (84 MPa), followed by Alternative 1 in second place (82.7 MPa), Alternative 3 in third place (80.2 MPa), Alternative 4 in fourth place (79.7 MPa), and Alternative 5 in fifth place (78.8 MPa). This indicates that for the high-strength Alternative 2, the TOPSIS Linguistic scale assigned very high importance (5 points), while for the low-strength Alternative 2, it assigned very low importance (1 point). 

The assessment of flexural performance heavily relies on the ultimate flexural strength. Table 6 displays the average ultimate flexural strength values for each specimen. Additionally, Table 6 includes linguistic terms corresponding to these average values. The analysis indicates that Sample 2 exhibits superior performance in terms of ultimate flexural strength.

In the tested alternatives, alternatives 2 demonstrated the highest strength at 148.9 MPa, followed by alternatives 1 at 147.4 MPa, alternatives 3 at 144.06 MPa, alternatives 4 at 140.4 MPa, and alternatives 5 at 138.2 MPa. Consequently, the Fuzzy-AHP-TOPSIS linguistic scale assigned a high importance rating of 5 points to Alternative 2 for its high strength, while Alternative 5 received a low importance rating of 1 point due to its lower strength.

**Figure 6:**
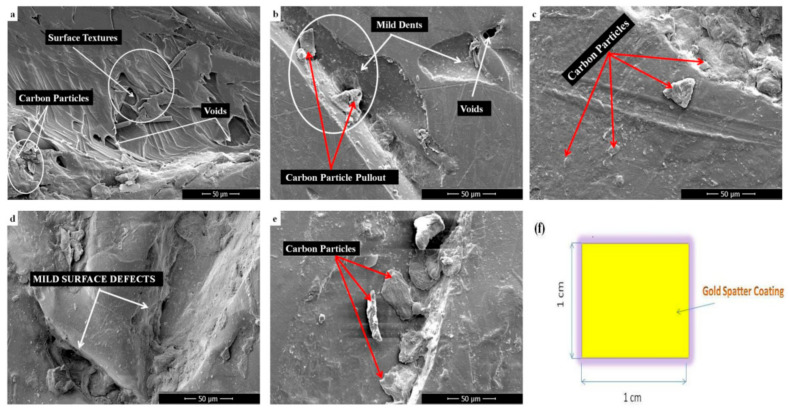
Morphology analysis of printed alternatives (**a**) A 1 (**b**) A 2 (**c**) A 3 (**d**) A 4 (**e**) A 5 (**f**) Geometric of gold-sputter-coated samples.

In terms of morphological outcomes, this study focuses on surface roughness and defects. Based on the morphological analysis, it was found that A5 exhibits fewer defects and less pronounced line pattern dents, attributed to the extruder melting temperature. Conversely, the surface of A1 shows defects and improper infill, also due to the extruder melting temperature. Consequently, A5 has been assigned a very high (VH) priority. In Figure 6a, the microstructure of Alternative 1 is presented, followed by the microstructures of Alternative 2 in Figure 6b, Alternative 3 in Figure 6c, Alternative 4 in Figure 6d, and Alternative 5 in Figure 6e. During FESEM analysis, the printed carbon-reinforced PEEK samples were coated with gold sputter, as depicted in Figure 6f. This process enhances the conductivity of non-conductive materials.

Alternative 5 exhibited a smooth surface finish with fewer defects compared to Alternative 3. Alternative 2 showed a slightly smoother surface finish with fewer lines than Alternative 4. However, Alternative 1 presented the roughest surface among the specimens, ranking fifth due to numerous surface flaws such as pores, gaps, and other imperfections. These rankings are consistent with the TOPSIS Linguistic Scale, which assigns very high importance (5 points) to Alternative 5 for its high strength and very low importance (1 point) to Alternative 1 for its lower strength. Table 7 illustrates the pair-wise matrix derived from observational data.

The fuzzified values of 5, 7, and 9 in the first column and first row of Table 8 represent Alternative 1 (Sample 1), which is deemed highly important (H) relative to Criteria 1.

Subsequently, the total number of individual values is normalized by dividing it by the maximum value of each column. Table 9 illustrates the FPIS (A^+^), which is derived by selecting the maximum value from each column representing beneficial criteria. This study employed useful criteria and morphological criteria to enhance mechanical properties. The subsequent step involves minimizing each column’s cost criterion (A^−^) to ascertain the fuzzy negative ideal solution.

Table 10 illustrates the distance of each alternative from the fuzzy positive ideal solution (FPIS), while Table 11 displays the distance of each alternative from the fuzzy ideal solution. Equation (2) below can be utilized to compute this distance [4].

d (ż,ẏ) = *sq*√((a_1_ − a_2_)^2^ + (b_1_ − b_2_)^2^+ (c_1_ − c_2_)^2^)) (2)

The FPIS is determined by selecting the value of a1 for each column if a_1_ represents the A^+^ value of each column and a_2_ represents the individual value of each column. Similarly, to calculate the fuzzy negative ideal solution (FNIS), the values of each column are substituted by A1 and A2. The resulting totals are presented in Table 11 and Table 12.

The decision matrix below was constructed after thoroughly analysing the criteria and alternatives. Table 12 displays the ranking of the alternatives based on the coefficient of closeness, as calculated by the formula employed.

Alternative 4 exhibited the highest coefficient of closeness compared to other samples, as indicated by the observations. The parameters of Alternative 4 demonstrated the highest mechanical properties among all samples for manufacturing impeller applications. The fourth set of parameters, comprising an infill density of 70%, a layer height of 0.15 mm, a printing speed of 60 mm/s, a platform temperature of 195 °C, an extruder temperature of 445 °C, and an extruder travel speed of 95 mm/s, were found to be the most suitable for CF-PEEK filament used in the production of rotating components.

Sensitivity analysis involves adjusting the weights of criteria to observe how the ranking of alternatives changes. This process helps ensure the reliability of the solutions and the ranking of alternatives by testing their robustness. Accordingly, altering the weight given to tensile and flexural properties does not change the ranking obtained. Similarly, increasing the weighting of tensile and surface defects also does not change the ranking. These sensitivity analyses confirm the correctness of the ranking, as it depicted in Table 13. Table 14 compares rankings between the PSI MCDM and fuzzy AHP-TOPSIS rating techniques. Maniya and Bhatt [47] used the preference selection index method to calculate the final preference score for each alternative by determining the preference value between each alternative and its variant. Overall, the comparative analysis has helped to elucidate the differences in rankings obtained from various MCDM techniques. In this case, the rankings from different MCDM techniques are similar to those from the fuzzy AHP-TOPSIS rating technique.

In this study, the validation of the data is based on the specific criteria applied, which aligns with previous research where tensile and flexural properties have been prioritized as key mechanical properties for impeller production. This approach ensures the validity of the selected criteria. The chosen process parameters are also validated, as previous researchers commonly employ them to determine suitable process parameters in FDM printing, which directly impact the mechanical properties of the printed parts. The selected optimization tool for decision-making is highly suitable, offering a slightly simpler procedure compared to other MCDM tools without compromising reliability. Furthermore, the research model has been validated by experts in the MCDM field, adding an additional layer of credibility to the study.

## 4. Conclusions

Various studies have explored the selection of process parameters in additive manufacturing. However, this research specifically focuses on determining the suitable process parameters for manufacturing rotating components using carbon-reinforced PEEK polymer, incorporating new and innovative assumptions. Unlike conventional approaches that rely on maximization or minimization after multi-stage optimization, this study employs a multicriteria decision-making method. This method effectively selects optimal process parameters by considering multiple criteria and alternative parameter clusters. The mechanical properties are used as criteria, while different clusters of parameters serve as alternatives. Based on previous research findings regarding mechanical properties, the study identifies the best process parameters from five different samples, ranging from the minimum to the maximum values for the selected material. Moreover, the criteria are selected based on the application of rotating components, ensuring the importance of these criteria and alternatives in the final product production, as validated by previous studies. Furthermore, the model developed in this research is tested and validated on another material with the same process parameters. Sensitivity analysis confirms the accuracy and consistency of the decision-making and optimization models. For instance, when assigning equal importance to all criteria, Alternative 4 is identified as suitable for carbon fiber PEEK. Even when different weight ages are assigned to criteria, this result remains unchanged, demonstrating the model’s reliability. Future researchers can use similar approaches to conduct selection and optimization studies with different materials or alternative multicriteria decision-making methods.

## Figures and Tables

**Table 2:** Scale conversion using Triangular Membership Function [4,5].

Saaty Parameters	Saaty Scale	Fuzzified Using Triangle Membership Function	
Equal consideration	1	1,1,1	
Moderate consideration	3	2,3,4	
Strong consideration	5	4,5,6	
Very strong consideration	7	6,7,8	
Extremely strong consideration	9	9,9,9	
Intermediate consideration	2	1,2,3	
	4	3,4,5	
	6	5,6,7	
	8	7,8,9	

**Table 3:** Terms related to linguistics and fuzzy numbers.

Linguistics Terms	Fuzzy Numbers Based on Triangular Membership Function	Linguistics Scales
Very Low (VL)	1,1,3	1
Low (L)	1,3,5	2
Average (A)	3,5,7	3
High (H)	5,7,9	4
Very High (VH)	7,9,9	5

**Table 4:** Observation of Young’s Modulus (in GPa).

	A1	A2	A3	A4	A5
A	3.61	3.25	3.71	3.05	3.17
B	3.54	3.21	3.64	2.99	3.08
C	3.49	3.11	3.69	3.01	3.01
Average	3.54	3.19	3.68	3.01	3.08
Importance	H	A	VH	VL	L

**Table 5:** Observation of UTS (in MPa).

	A1	A2	A3	A 4	A5
A	82.1	83.7	80.2	80.3	79.8
B	82.6	84.2	80.6	79.6	78.6
C	83.4	84.1	79.8	79.2	78.2
Average	82.7	84	80.2	79.7	78.8
Importance	H	VH	A	L	VL

**Table 6:** Observation of Ultimate Flexure Strength (in MPa).

	A1	A2	A3	A4	A5
A	148	149.6	144.5	140.3	138.6
B	147.3	148.2	143.6	141.2	137.9
C	146.9	148.9	144.1	139.7	138.1
Average	147.4	148.9	144.06	140.4	138.2
Importance	H	VH	A	L	VL

**Table 7:** Linguistic Scale of Evaluation matrix.

	C1	C2	C3	C4
Alternative 1	H	H	H	VL
Alternative 2	VH	A	VH	A
Alternative 3	A	VH	A	H
Alternative 4	L	VL	L	L
Alternative 5	VL	L	VL	VH

**Table 8:** Fuzzified Evaluation Matrix.

		C1			C2			C3			C4	
Alternative 1	5	7	9	5	7	9	5	7	9	1	1	3
Alternative 2	7	9	9	3	5	7	7	9	9	3	5	7
Alternative 3	3	5	7	7	9	9	3	5	7	5	7	9
Alternative 4	1	3	5	1	1	3	1	3	5	1	3	5
Alternative 5	1	1	3	1	3	5	1	1	3	7	9	9

**Table 9:** Compute A^+^ and A^−^.

	C1			C2			C3			C4		
**A1**	0.555	0.777		0.555	0.777		0.555	0.777		0.111	0.111	0.333
			1			1			1			
**A2**	0.777			0.333	0.555	0.777	0.777			0.333	0.555	0.777
		1	1					1	1			
**A3**	0.333	0.555	0.777	0.777			0.333	0.555	0.777	0.555	0.777	
					1	1						1
**A 4**	0.111	0.333	0.555	0.111	0.111	0.333	0.111	0.333	0.555	0.111	0.333	0.555
											
**A 5**	0.111	0.111	0.333	0.111	0.333	0.555	0.111	0.111	0.333	0.777		
											1	1
**A^+^**							0.777			0.777		
	0.777	1	1	0.777	1	1		1	1		1	1
**A^−^**		0.111			0.111	0.333	0.111	0.111	0.333	0.111	0.111	0.333
	0.111		0.333	0.111								

**Table 10:** Compute the FPIS (A^+^).

	C1	C2	C3	C4	di^+^
Alternative 1	0	0.181	0	0.748	0.929
Alternative 2	0	0.384	0	0.384	0.769
Alternative 3	0.384	0	0.384	0.181	0.951
Alternative 4	0.379	0.748	0.601	0.601	2.331
Alternative 5	0.748	0	0.748	0	1.496

**Table 11:** Compute the FNIS (A^−^).

	C1	C2	C3	C4
Alternative 1	0.601	0	0.601	0
Alternative 2	0	0.384	0.748	0.384
Alternative 3	0.384	0.748	0.384	0.601
Alternative 4	0.181	0	0	0
Alternative 5	0	0.181	0	0.748

**Table 12:** Decision Matrix.

	Closeness Co-Efficient (Cci)	Rank
Alternative 1	0.564	**III**
Alternative 2	0.663	**II**
Alternative 3	0.060	**V**
Alternative 4	0.722	**I**
Alternative 5	0.383	**IV**

**Table 13:** Sensitivity Assessment.

	Set 1—High Weightage to C1, C2	Set 2—High Weightage to C1, C2, C3	Set 3—High Weightage to C1, C2, C4	Set 4 (Original)—Equal Weightage to All Criteria	Rank
Alternative 1	0.589	0.498	0.509	0.564	**III**
Alternative 2	0.699	0.572	0.589	0.663	**II**
Alternative 3	0.092	0.049	0.054	0.060	**V**
Alternative 4	0.813	0.633	0.676	0.722	**I**
Alternative 5	0.412	0.282	0.302	0.383	**IV**

**Table 14:** Comparative Analysis Using the Preference Selection Index Method.

	Preference Selection Index (PSI) Method	Fuzzy-AHP TOPSIS (This Method)	Rank
Alternative 1	0.522	0.564	**III**
Alternative 2	0.654	0.663	**II**
Alternative 3	0.085	0.060	**V**
Alternative 4	0.746	0.722	**I**
Alternative 5	0.392	0.383	**IV**

## Data Availability

Data are contained within the article.
